# SET-LRP and cellulose fiber: a versatile platform to tune the surface properties of cellulose

**DOI:** 10.1039/d6ra03700c

**Published:** 2026-07-06

**Authors:** Enguerrand Barba, J. Benedikt Mietner, Dhanya Raveendrana, Benedikt Sochor, Sarathlal Koyiloth Vayalil, Stephan V. Roth, Julien R. G. Navarro

**Affiliations:** a Institute of Wood Science, Universität Hamburg Hamburg Germany julien.navarro@uni-hamburg.de; b Deutsches Elektronen-Synchrotron DESY Notkestrasse 85 22607 Hamburg Germany; c Advanced Light Source, Lawrence Berkeley National Laboratory 6 Cyclotron Rd Berkeley CA 94720 USA; d Applied Science Cluster, UPES Dehradun Uttarakhand 248007 India; e KTH Royal Institute of Technology, Department of Fibre and Polymer Technology Teknikringen 56-58 10044 Stockholm Sweden

## Abstract

Cellulose fibers are very promising additives for polymer composites for reinforcing. However, they suffer from poor compatibility with most polymer matrices due to their hydrophilic nature. In this article, we will expand on a previously developed method for improving this compatibility consisting in grafting an acrylate monomer to the surface of cellulose fibers using SET-LRP. This time, we will investigate its versatility by using several selected acrylates (butyl acrylate, stearyl acrylate, docosyl acrylate and di(ethylene glycol) ethyl ether acrylate) as well as styrene. In order to understand the different effects of this monomer variation, SAXS, DSC, FESEM and contact angle measurements were performed on the composites with ABS and the final products were 3D printed by Fused Deposition Modeling (FDM).

## Abstract 2

5 different monomers were grafted on the surface of cellulose fibres by SET-LRP. Their compatibility with an ABS matrix and the printability and physical properties of the resulting composite were then assessed.

## Graphical abstract

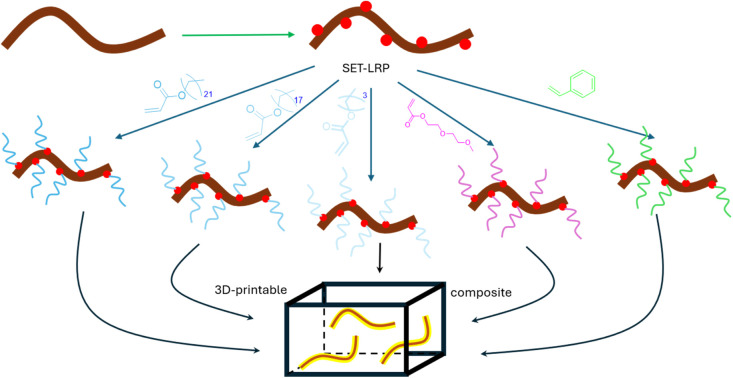

## 1 Introduction

3D printing, also known as additive manufacturing, is a category of manufacturing technologies that produces objects by stacking layers of materials on top of each other. By ensuring the adhesion of each layer with the adjacent layers, a 3D object can be made. This can be done with a great variety of materials, such as metals (powder bed fusion,^1^ direct energy deposition,^2^ binder jetting,^3^ bound powder extrusion^4^), gels (direct ink writing^5^), resins (stereolithography^6^), thermoplastics (fused deposition modeling, selective laser sintering^7^), and even concrete.^8^ Thanks to this great diversity, 3D printing has seen uses in many fields: concrete printing is used to print buildings^9,10^ or bridges,^11^ metal printing techniques are the one with the widest presence in industry,^12,13^ while gels are promising for pharmaceutical applications due to their biocompatibility.^14,15^ On the other hand, because of their ease of use, resins and thermoplastics are used not only in an industrial context,^16,17^ but are also the main printing methods accessible to the general public.

Among these techniques, Stereolithography and Fused Deposition Modeling (FDM) are the most accessible to the general public, with FDM being the more widespread, due to its ease of use. FDM uses a filament of thermoplastic which, after being melted through a nozzle, is used to print thin lines on a printing bed, thereby forming a thermoplastic layer. By printing layer upon layer, a 3D object can be made. There is a great variety of filaments available on the market for printing, with some of the most common ones being based on PLA, TPU, PET, Nylon or ABS.^18,19^ Some additives can also be added to the matrix to control their printability, their color and other mechanical or thermal properties of the filament. These properties are especially important, as they determine the final properties and aspects of the printed object.

Cellulose fibers are very promising additive:^20,21^ they are cheap, readily available, have high tensile strength^22^ and aspect ratio, good biocompatibility, and are extracted from renewable resource (such as wood).^23^ Their addition to 3D-printing filaments can bring a wide variety of advantages, reducing the total proportion of petrochemical materials in the composite and promoting the recycling of wood wasted (as is common with WPCs^24,25^), together with various improvement to the filament's printability with improved dimensional stability and reduced warping from its use as filler^26^ and to its mechanical properties with improvements to the polymer's Young's modulus^26^ through the reinforcement effect. Their main drawback, however, comes from their hydrophilic nature. As most thermoplastics are hydrophobic, this leads to poor fiber-matrix compatibility, which not only prevents the expected reinforcing effect, but even weakens the mechanical properties of the composite due to the lack of adhesion between the two elements.

In a previous article, we outlined a surface modification of cellulose using SET-LRP to graft hydrophobic monomers on the surface of cellulose and showed it increased in compatibility with HDPE,^26^ while another one showed that new properties can be added to the cellulose through selection of the monomer, such as antibacterial properties. However, these articles only highlight specific modifications, and lack the versatility needed to make this method widely applicable.

In the following article, surface modifications of cellulose fibers were performed by grafting butyl acrylate (BA), stearyl acrylate (SA), docosyl acrylate (DA), di (ethylene glycol) ethyl ether acrylate (DEGEEA), and styrene (St). The studied products show both the versatility of this method and the tunability of the modified fiber's properties, such as hydrophobicity or thermal properties.

## 2 Experimental section

### 2.1 Materials

DMSO at 99%, *N*,*N*-dimethylformamide (DMF) at 99%, imidazole (99%), 2-bromoisobutyric acid (98%), 2-(dimethylamino)ethyl acrylate (98%) stab. with *ca* 0.1% 4-methoxy-phenol, 1-bromododecane (98%), and the copper wire (1.0 mm diameter, annealed, 99.9% (metal basis) were purchased from Alfa Aesar. 2-Propanol at 99.5% was purchased from J. T. Baker. The cellulose flour CW 630 PU/ARBOCEL was thankfully donated by JRS Rettenmaier. The cellulose fibers were produced using ECF (elemental chlorine-free) bleached kraft softwood pulp from MERCER Stendal GmdH. Poly(vinyl chloride) (PVC) low molecular weight, CDI (reagent grade) and octadecyl acrylate (contains 200 ppm monomethyl etherhydroauinone as inhibitor, 97%) were procured from Sigma-Aldrich. Processing agents used for PVC extrusion were Mark CZ2000 from Galata Chemicals, Paraloid K125 ER from Dow Chemical, Loxiol G60 and Loxiol G21 from Emery Oleochemicals, Ligalub GT from Peter Greven GmdH and Licocene PE4201 from Clariant.

### 2.2 Method

#### 2.2.1 Preparation of cellulose fiber (CF)

Cellulose fibers (CF) were obtained by milling 10 g of a cellulose sheet with 200 mL of distilled water for 5 min at 1500 rpm in a Retsch RS200 grinding machine. The resulting fiber water suspension was then solvent exchanges to DMSO by adding DSMO until a 1 : 1 DMSO:water ratio was reached, centrifugation at 6000 rpm (4427 g), then redispersing in DMSO and centrifuging the solution 2 more times.

#### 2.2.2 Synthesis of the CF-based macro-initiator (CF-MI)

Macro-initiator was synthesized as described in a previous papers.^26^ Briefly, 0.1 g of cellulose were dispersed in 7 mL of DMSO. The solution was then degassed with N_2_, heated at 55 °C and 3 g of imidazole were added to it. At the same time, 4 g of 2-bromo-isobutyric acid were dissolved in 20 mL of DMSO. This solution was also degassed with N_2_, then 4 g of CDI were slowly added to it, to avoid excessive gas production. The solution was then left under N_2_ for 30 minutes to 1 hour, until the gas emission stopped. The 2-bromo-isobutyric acid solution was then slowly poured in the cellulose solution. The resulting solution was degassed with N_2_, then left to react overnight under stirring. Once the reaction was finished, it was centrifuged at 6000 rpm (4427 g) for 20 minutes, then washed by 8 cycles of dispersion in DMSO followed by precipitation by centrifugation (20 minutes, 6000 rpm (4427 g)) and supernatant removal.

#### 2.2.3 Procedure for grafted monomers on cellulose by SET-LRP

100 µL of Me_6_-TREN (3.74 M) were dissolved in 5 mL of DMSO under Schlenk conditions to form the ligand solution. In a flask, 0.1 g of macroinitiator gel (dry mass) were dispersed in 15 mL of DMSO under nitrogen atmosphere.

The monomer was then dissolved in 15 mL of solvent (see table below) under gentle stirring. After complete dissolution of the monomer, it was passed through a basic aluminum oxide column to remove the stabilizing agent, and 15 mL of DMSO were used to wash as much of the remaining monomer as possible from the column. Both the monomer and the DMSO were recovered in the flask containing the cellulose fiber suspension. The flask was once again degassed with N_2_ and kept under nitrogen atmosphere.

6 cm of copper wire was coiled in a spring-like shape, then put in a concentrated HCl for a few minutes. The wire was then washed with distilled water and immersed in acetone. After a few minutes, the wire was dried, before adding it to the reaction flask.

Finally, the solution was heated at 40 °C and the ligand solution was added (Table 1). The reaction was left to react overnight (16 hours).

**Table 1:** Synthesis condition for the SET-LRP with butyl acrylate (BA), stearyl acrylate (SA), docosyl acrylate (DA), styrene (St) and di(ethylene glycol) ethyl ether acrylate (DEGEEA)

Monomer	Mass/volume	Monomer solvent	Ligand (Me_6_-TREN) solution
Butyl acrylate (BA)	2.2 g	Toluene	0.2 mL
Stearyl acrylate (SA)	5 g	Toluene	0.2 mL
Docosyl acrylate (DA)	5.85 g	Toluene	0.2 mL
Styrene (St)	1.8 mL	DMSO	0.4 mL
Di (ethylene glycol) ethyl ether acrylate (DEGEEA)	2.85 mL	DMSO	0.2 mL

After the reaction, the product was left to cool at room temperature and started to for a solid block. The liquid phase was removed, and the block was dissolved in the monomer solvent, then centrifuged at 6000 rpm (4427 g) for 1 h. The solid phase was redissolved in the monomer solvent, then centrifuged at 6000 rpm (4427 g) for 1 h. This process was repeated 2 times to purify the grafted cellulose.

#### 2.2.4 Thin film preparation

Thin films of modified and unmodified fibers were made by film casting the fibers in toluene.

The composite films in ABS were made by film casting of 0.45 g of ABS and 0.05 g of filler (modified and unmodified cellulose fibers and flour) in toluene. In both cases, all products were stirred overnight to ensure full dissolution, poured in an aluminum plate and left to dry overnight. Once the solvent was evaporated, the film was put in a vacuum dryer for 2 h to remove the last traces of solvent.

#### 2.2.5 Composite mixing

The extrusion was carried out using a Haake Minilab 3 extruder. The extrusion temperature was set to 230 °C, with a 35 rpm screw rotation speed, using counter-rotating screws.

#### 2.2.6 3D-printing

The printing was performed with a nozzle temperature of 230 °C and bed temperature of 100 °C. The nozzle temperature was increased by 5 °C and the extrusion multiplier increased by 0.1 to print composites.

### 2.3 Analysis

FTIR (Fourier Transform InfraRed spectroscopy) was performed using Bruker Vector 33 FTIR Fourier-transform infrared Spectrometer I18500 PS15. Spectrums were recorded with 64 scans in the spectral region on 3800–450 cm^−1^. The spectral resolution is 4 cm^−1^.

DSC (Differential Scanning Calorimetry) was performed using a DSC Mettler Toledo/DSC 3+ Starsystem. Each sample was dried overnight under vacuum before being scanned for 3 cycles under N_2_ from 25 °C to 250 °C.

Contact angles measurements with water were performed with a Krüss DSA100 using the Krüss Advance software. The value considered was the average of both angles measured from a drop on the surface of a thin film made of the measured material.

SAXS measurements were done at the beamline P03 of PETRA III at the DESY synchrotron at Hamburg.^27^ Samples were ABS-only film and 10 w/w% of modified fibers in ABS films, prepared by film casting. The beam wavelength was *λ* = 1.0330 Å, and a sample-to-detector distance (SDD) of 9407 mm. 2D patterns were recorded with a PILATUS 2 M detector (Dectris, Switzerland) with a pixel size of 172 µm. Each measurement was performed by scanning the sample following a 3 × 3 grid with a distance of 1 mm between each point for ABS or a 11 × 11 grid with a 0.2 mm between each point for the composites, and an acquisition time of 1 s to avoid beam damage. Images were then summed and radially integrated to obtain a *I*(*q*) curve. These curves were fitted using the lmfit python library and SASView and the extended Guinier–Porod model with additional terms for fiber ordering (Lorenzian function) and crystallinity (Guassian function) (see SI).

## 3 Result and discussion

**Scheme 1:**
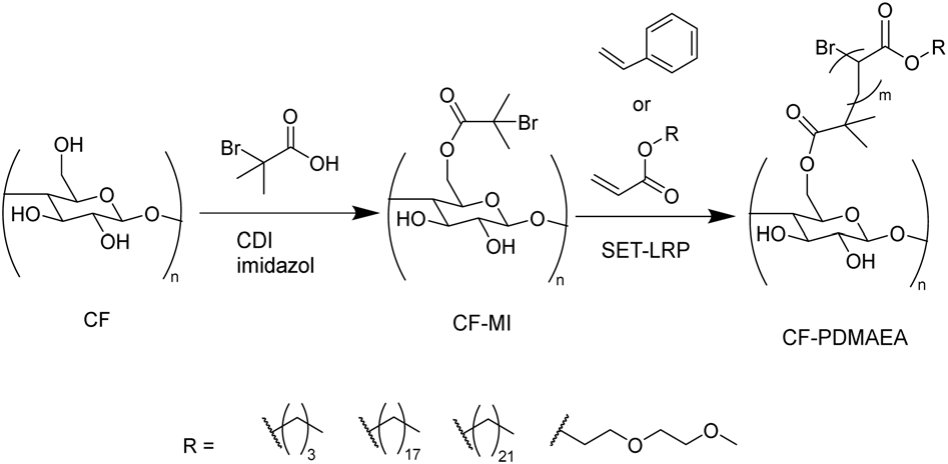
Synthetic process for the production of CF-MI and graft polymerization of the CF-based macroinitiator with the various monomers *via* SET-LRP. The grafting was shown at a C6 position as an example.

The first step was the synthesis of cellulose based macro-initiator (CF-MI). Cellulose fibers are reacted with 2-bromoisobutyric acid in order to attach bromines to its surface, making it suitable as initiator for the next step. SET-LRP was then performed using a selection of monomers: poly(butyl acrylate) (PBA), poly(stearyl acylate) (PSA), poly(docosyl acrylate) (PDA), poly(di(ethlyen glycol) ethly ether acrylate) (PDEGEEA) and poly(styrene) (PSt) as exposed in Scheme 1.

**Fig. 1:**
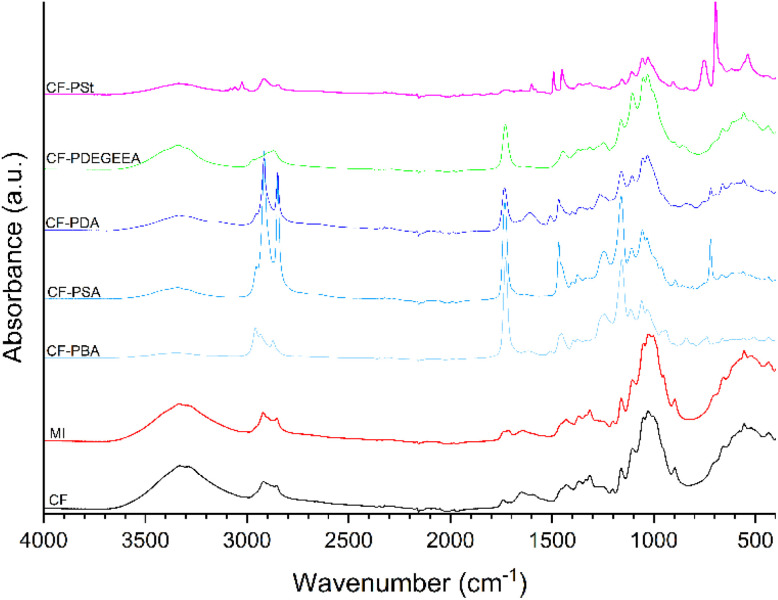
IR spectra of (from bottom to top): cellulose fibers (CF), macro-initiator (MI), CF grafted with: poly(butyl acrylate) (PBA), poly(stearyl acylate) (PSA), poly(docosyl acrylate) (PDA), poly(di(ethlyen glycol) ethly ether acrylate) (PDEGEEA) and poly(styrene) (PSt).

The good proceeding the each step was checked by IR. The successful grafting of the initiator on the surface of cellulse can be seen from the increase of the peak around 1722 cm^−1^, representing the C=O double bound stretching. A further increase of this peak is observed with the polymerization of the 4 acrylates, as this double bound is also found in them. This, together with the confirmation by ^13^C NMR of the polymer grafting in similar conditions performed in previous works,^26^ was used as proof of the success of polymerization. Polymerization with styrene, however, showed no such increase. Instread, two sharp and intense peak around 698 and 758 cm^−1^ appeared, due to the aromatic C–H bending, showing the success of the polymerization (Fig. 1).

### 3.1 DSC analysis

DSC analysis of each grafted fiber was then performed to assess the thermal properties of the modified fibers and their impact on the composite with ABS.

**Fig. 2:**
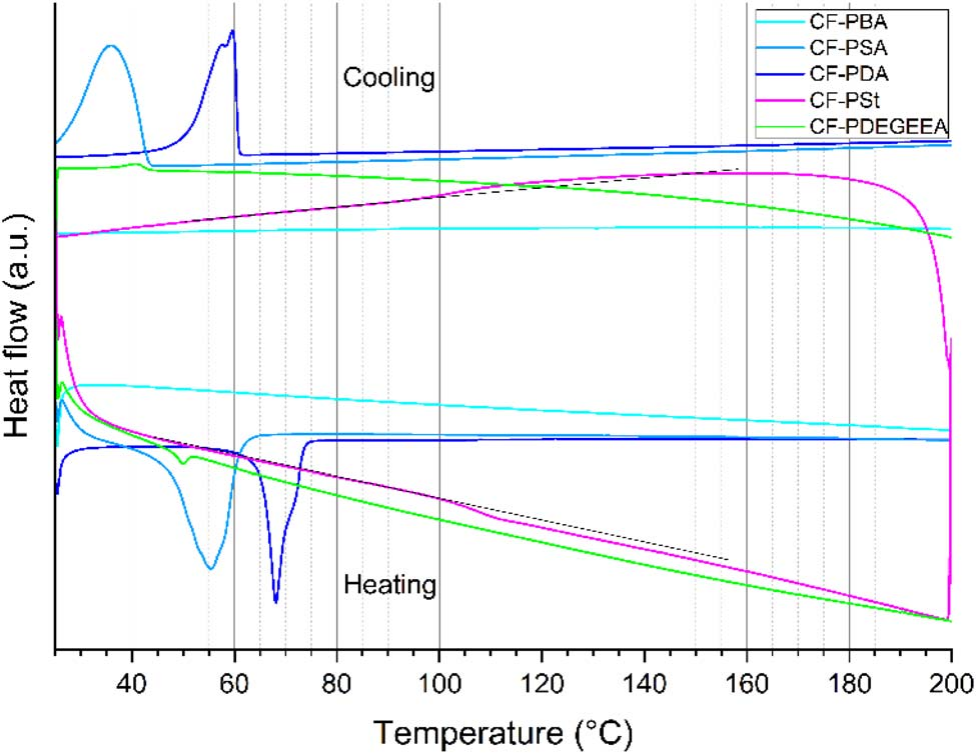
DSC results of CF-PBA, CF-PSA, CF-PDA, CF-PSt and CF-PDEGEEA. Tangents were added to the CF-PSt plots for clarity.

From the DSC of each individual monomer in Fig. 2, some features of their thermal profile can be seen: CF-PSA and CF-PDA both exhibit strong peaks, with CF-PSA having its melting and reverse freezing around 43 °C, and CF-PDA having its around 62 °C. This highlights the influence of the monomer chain length, as a 4-carbon difference led to a 19 °C change in the glass transition. Following this logic, CF-PBA did not show any phase change in the measured range. The fact that it has already passed its glass transition temperature is corroborated with its rubbery texture at room temperature. CF-PSt shows no peak in addition to its glass transition and reverse glass transition, but only the change of baseline characteristic of a different phase. Both phase transitions occur around 108 °C. Finally, CF-PDEGEEA exhibits small peaks for melting and freezing, at 47 °C and 43 °C respectively (Table 2).

**Table 2:** DSC results of CF-PBA, CF-PSA, CF-PDA, CF-PSt and CF-PDEGEEA

	Melting & glass transition (°C)	Solidification & reverse glass transition (°C)	Carbon chain length
CF-PBA	—	—	4
CF-PSA	43	43	18
CF-PDA	62	61	22
CF-PSt	108	108	—
CF-PDEGEEA	47	43	—

**Fig. 3:**
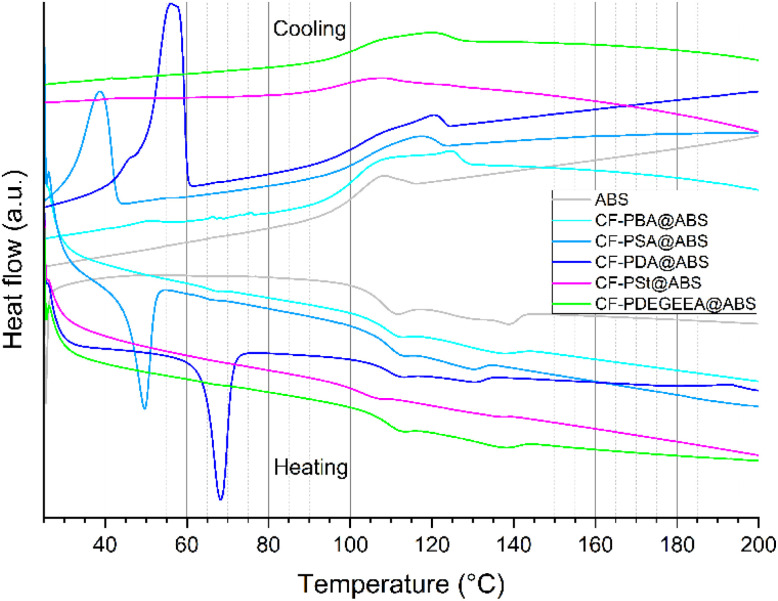
DSC result of CF-PBA, CF-PSA, CF-PDA, CF-PSt and CF-PDEGEEA compounded in ABS.

DSC of ABS and its composite were then made to see the influence of the modified fibers on the composite thermal properties (Fig. 3). A common feature of all composites except CF-PSt@ABS was an increase in the width and starting temperature of the reverse glass transition: instead of a single peak around 116 °C, the freezing of the composites have the shape of a broad band or a pair of peaks starting around 123 to 128 °C and ending between 200 to 205 °C. This was attributed to the fibers forcing some structure at their interface with the matrix. This also explains why CF-PSt@ABS didn't show this effect, as styrene is one of the base monomers of ABS, and as such wouldn't create as strong an interface as the other monomers grafted on cellulose.

CF-PSA and CF-PDA both added strong peaks for their respective melting and freezing, with only CF-PSA shifting to lower temperatures. Similarly, the thermal properties of CF-PBA were also directly translated to the composite, with no additional peaks being displayed. CF-PDEGEEA's freezing peak is barely visible, which can be expected since it only represents 10% w/w of the composite. Its main influence on thermal properties seems to be the change in the ABS reverse glass transition to higher temperature. Finally, CF-PSt's thermal properties could not be seen from the DCS due to its phase transition, which shows no peak, occurring at the same temperature as ABS. This is not surprising, as styrene is one of the three monomers forming ABS. It does, however, seem to lower the intensity of the ABS phase transition peaks. This could be due to a strong impact of the modified fibers on the matrix's structure, indicating a good compatibility (Table 3).

**Table 3:** DSC result of CF-PBA, CF-PSA, CF-PDA, CF-PSt and CF-PDEGEEA compounded in ABS

	Melting & glass transition (°C)	Solidification & reverse glass transition (°C)	Carbone chain length
ABS	100 &135	116	Matrix
CF-PBA	—	—	4
CF-PSA	43	43	18
CF-PDA	60	61	22
CF-PSt	100	100	—
CF-PDEGEEA	—	—	—

### 3.2 SAXS measurements

**Fig. 4:**
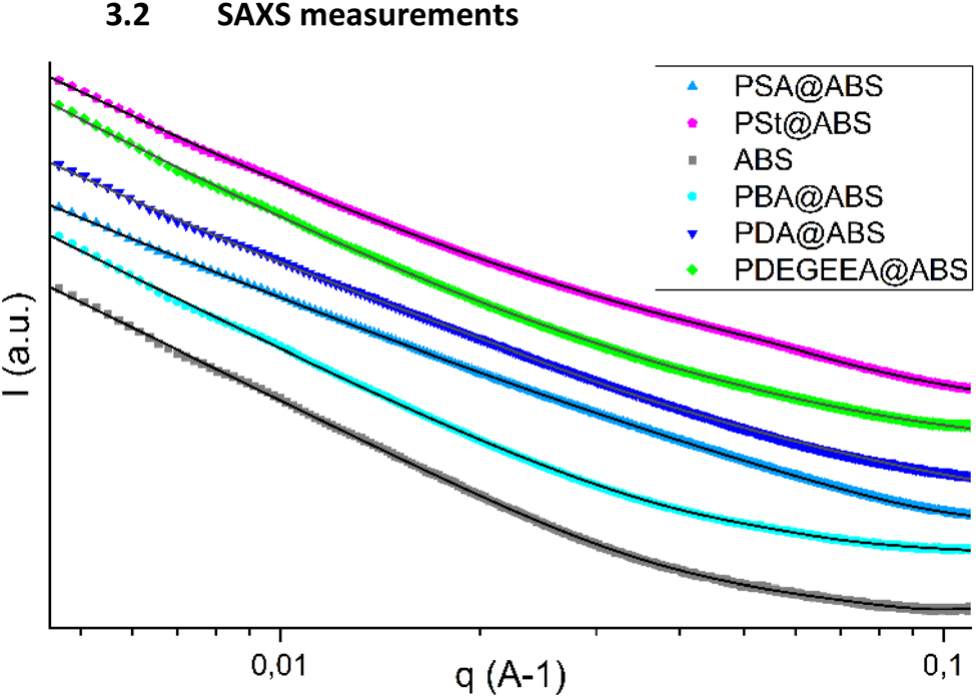
SAXS data and their respective fits for ABS, CF-PBA@ABS, CF-PSA@ABS, CF-PDA@ABS, CF-PDEGEEA@ABS and CF-PSt@ABS films.

In order to strengthen the understanding of the structure of the polymer modified CF encapsulated in the ABS matrix, a series of SAXS measurements are performed. A common feature of the SAXS spectra of ABS and its composites is an undulation at low *q*, which can't be fitted with the Guinier–Porod model. This indicates a large-scale ordering in ABS that remains unchanged in composites (Fig. 4).

An increase of the Porod exponent is immediately visible between ABS and CF-PDEGEEA@ABS, which shows both the stronger influence of the grafted polymer compared to cellulose fibres (as the contribution of the latter would lower the Porod exponent due to the more unfolded structure of the micro-fibrils), and the structure of the grafted PDEGEEA chains as spherical bundles. Similarly, one can compare the values of CF-PSA@ABS and CF-PDA@ABS. As the structure of their monomer is very close, little difference is expected in their arrangement, however, a wide gap between their Porod exponent (3.23 for CF-PDA@ABS and 2.99 for CF-PSA@ABS) shows the better mixing of CF-PSA compared to CF-PDA. Finally, taking in consideration the good mixing observed for both CF-PBA@ABS and CF-PSt@ABS, one can see from their Porod exponent that their grafted polymer is less unfolded than PSA and PDA, with PBA being the least unfolded.

The changes in fiber–fiber correlation distance *ξ* can be interpreted as indicative of the change in the distance between grafted polymer chains, the value of ABS alone being indicative of the matrix's inter-polymer distance and the composite's value being influenced by both the matrix and the grafted polymer. This trend follows the side chain length for PBA, PSA and PDA, and sets PDEGEEA as having the highest inter-fiber distance. However, these values should be taken with caution due to the influence of a Gaussian in the same region of the spectra, which can't be accurately fitted because most of it still remains outside the measured range.

### 3.3 Contact angle measurement

Films made of each modified fiber, as well as films of ABS and films of each modified fiber in ABS (composites with a 10 w/w% ratio) were made to measure their contact angles.

**Fig. 5:**
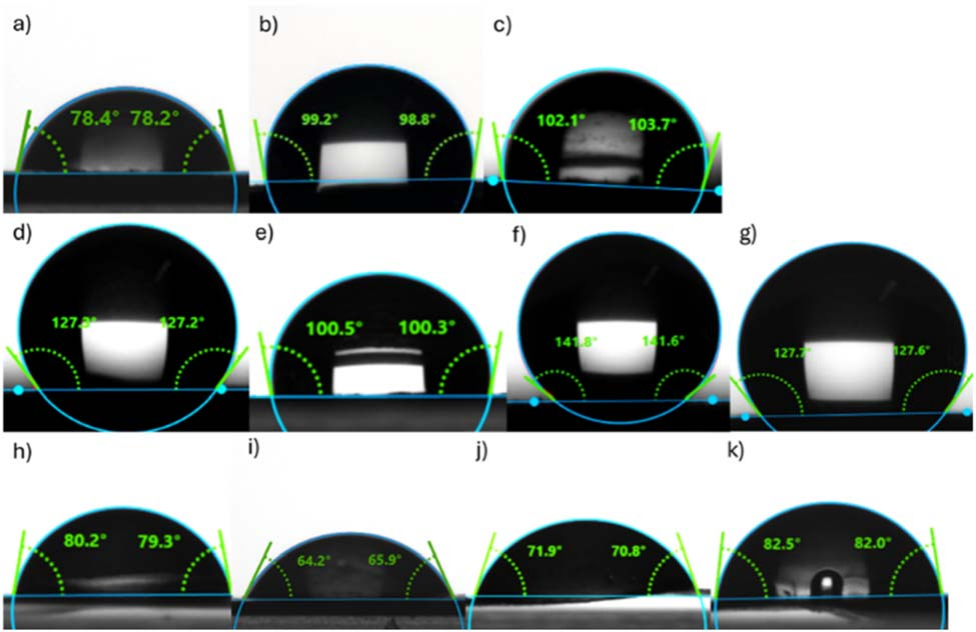
Contact angle measurement of (a) ABS, (b) CF-PBA, (c) CF-PBA@ABS, (d) CF-PSA, (e) CF-PSA@ABS, (f) CF-PDA, (g) CF-PDA@ABS, (h) CF-PDEGEEA, (i) CF-PDEGEEA@ABS, (j) CF-PSt and (k) CF-PSt@ABS. The liquid used for the drop was water.

The first thing to be noticed is the wide range of values for the contact angles seen on Fig. 5 and Table 4, showing that the surface modification was very effective at changing the surface properties of the fibers. The contact angle values went from 21° for unmodified cellulose to values close to that of the homopolymer: 99° for CF-PBA compared to 65° to 77° in the litterature,^28,29^ 127° for CF-PSA compared to 117° found in literature^30^ and 71° for PSt compared to values in the range of 75° to 84° in literature.^31,32^ While PDA and PDEGEEA don't seem to be well referenced in literature, similarly high values could be found for PDA used as coating material, with value reaching 140° when coating cotton.^33^ This same article highlights the higher values obtained for PSA as coating compared to neat PSA, further confirming the efficiency of our method.

**Table 4:** Main fitting parameters for the SAXS measurements of ABS, CF-PBA@ABS, CF-PSA@ABS, CF-PDA@ABS, CF-PDEGEEA@ABS and CF-PSt@ABS films. Detailed fitting parameters and error are shown in the SI

	ABS	CF-PBA@ABS	CF-PSA@ABS	CF-PDA@ABS	CF-PDEGEEA@ABS	CF-PSt@ABS
*n*	3.70	3.61	2.99	3.23	3.78	3.40
*x*_0_	OOB	OOB	OOB	OOB	OOB	OOB
*ξ*	9	17	32	59	66	39

In addition to this, the composite also had some very important shifts in hydrophobicity, despite the loading on fiber being only 10 w/w%. All 3 composited with CF-PBA, CF-PSA and CF-PDA had contact angle values above 100°, with CF-PDA@ABS reaching 128°. This represents an increate of 20° to 50° compared to the neat composite. The effect of compounding was far less visible for the other 2 monomers, as their contact angles were already close to that of ABS.

Both observations clearly showcase the success of the method in tuning the fibers properties.

In addition to this, the contact angle values give some explanations on the SAXS measurements. The high disturbance of CF-PSA and CF-PDA on the composite structure can be attributed to their very high hydrophobicity (127° and 141° respectively) compared to the matrix (78°, which is slightly lower that values reported in literature^34^). All other modified fibers had hydrophobicity comparable to that of ABS (Table 5).

**Table 5:** List of contact angles values of ABS, CFfibers-PBA, CFfibers-PSA, CFfibers-PDA, CFfibers-PDEGEEA, CFfibers-PSt and their composites made of 10 w% modified cellulose in ABS

	Sample	Composite with ABS
ABS	78.3°	—
CF	21.5°	—
CF-PBA	99.5°	103°
CF-PSA	127°	100.4°
CF-PDA	141.5°	128°
CF-PDEGEEA	70.8°	73° to 65° (fast decrease)
CF-PSt	71.3°	82°

### 3.4 FESEM imaging

FESEM images of the samples were then taken to investigate the structure of the composites. The FESEM samples were made by breaking a filament of the composite used for printing and by analyzing the broken cross-section.

**Fig. 6:**
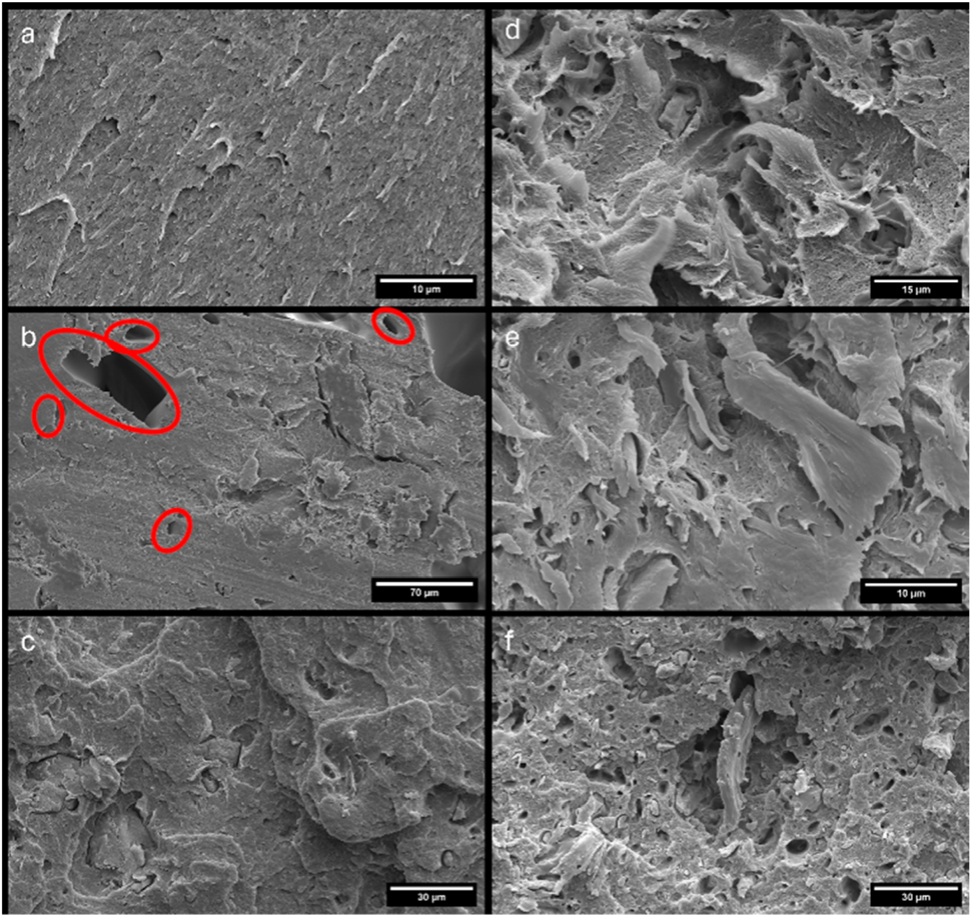
FESEM pictures of (a) ABS, (b) CF-PDEGEEA@ABS, (c) CF-PSt@ABS, (d) CF-PBA@ABS, (e) CF-PSA@ABS and (f) CF-PDA@ABS. Pull-out were indicated in red for CF-PDEGEEA.

The first noticeable thing in these pictures is that CF-PDEGEEA@ABS has a very smooth breaking surface like ABS, while all other composites have a much rougher surface. In addition to this, CF-PDEGEEA shows many pull-outs (in red in Fig. 6b) with very smooth inner surface, and no fibre can be observed remaining in the composite's surface. This demonstrates the poor fibre-matrix compatibility of this sample.

Other sample's breaking surface had a much rougher surface than ABS, showing that the fiber-matrix interaction was stronger that for CF-PDEGEEA@ABS. CF-PDA@ABS (Fig. 6 picture f) showed a rough surface with some pull-out, but the majority of the fibers remained, showing a decent compatibility. The amount of pull-out seen decreased with CF-PSA@ABS and even more with CF-PBA@ABS (Fig. 6 pictures e and d), with the latter showing very little pull-out. This is consistent with the decreasing difference in hydrophobicity observed in contact angle measurements.

Finally, CF-PSt@ABS shows the best fiber-matrix compatibility: it showed no signs of pull-out and the gap between fibers and matrix was the smallest out of all observed samples. This high compatibility can be explained by the fact that styrene is one of the constituting monomers of ABS and would allow π–π stacking between the modified fibers and the matrix, in addition to hydrophobic interactions.

### 3.5 FDM printing

**Fig. 7:**
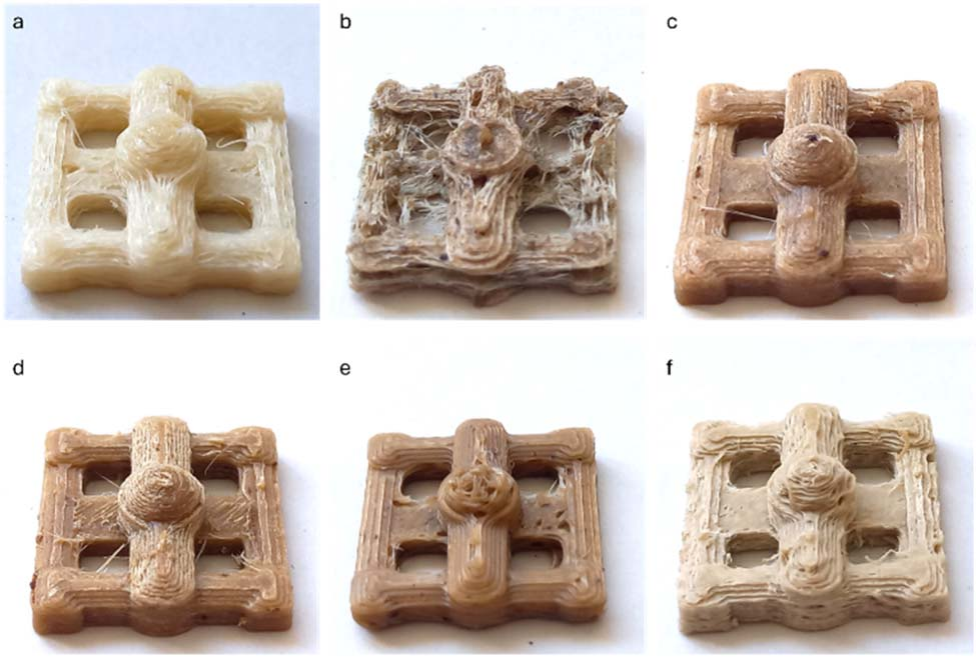
3D printed samples of (a) ABS, (b) CF-PDEGEEA@ABS, (c) CF-PSt@ABS, (d) CF-PBA@ABS, (e) CF-PSA@ABS and (f) CF-PDA@ABS.

Finally, all samples were printed using a FDM printer (Fig. 7).

The most obvious result of these prints is the poor print quality of CF-PDEGEEA@ABS. The filament was brittle, repeatedly breaking inside the printer, and made the printing process fastidious for an unprecise print. In this case, the modification of fiber clearly decreased the fiber-matrix compatibility.

However, other samples had better effects: the series of CF-PBA@ABS, CF-PSA@ABS and CF-PDA@ABS showed good printability, with CF-PBA@ABS having the best quality. This result is consistent with the composite analysis showing it to have the best fiber-matrix compatibility. CF-PSt@ABS showed the best printability of all samples, not with a near-perfect print obtained without any kind of complication. This is once again in line with the result of FESEM and SAXS analysis which highlighted the great compatibility of styrene with ABS. Another interesting result from these printings is the lower print quality improvement of CF-PSA@ABS compared to a the same modification in HDPE (CF-PSA@HDPE) done in a previous publication,^26^ showing the importance of being able to freely select the most adequate monomer for a given matrix.

In addition to this, all composites except for CF-PDEGEEA@ABS showed reduced stringing compared to pure ABS filament.

## 4 Conclusions

In this work, we modified cellulose fiber using the SET-LRP of five monomers: butyl acrylate (BA), stearyl acrylate (SA), docosyl acrylate (DA) and di (ethylene glycol) ethyl ether acrylate (DEGEEA). All modifications were successful and after analysis, it was shown that it greatly impacted the thermal properties and hydrophobicity of the fibers, leading to vastly different compatibilities with a given polymer matrix.

Previous articles showed this methods' ability to improve cellulose fibres' compatibility with both HPDE and PVC matrixes by choosing an appropriate monomer, and the possibility to transfer some of the monomer's properties to the whole composite, with the use of DMAEA as monomer. The results from this article highlight the versatility of this method as a synthetic platform for tuning the surface properties of cellulose fibers and its compatibility with a given polymer matrix by varying the chosen monomer in a third polymer matrix (ABS) without significant changes to the synthesis method. In addition to this, a first non-acrylate monomer was used in this work to shows that this synthetic platform isn't confined to this single monomer category. However, the analysis could not allow the quantifying and sorting the fibre-matrix of the monomers.

While many other methods have been developed for the surface modification of cellulose, such as its esterification,^35,36^ click chemistry,^37^ oxidation,^38^ silylation^39^ or urethanization,^35^ these methods usually either require very specific chemical function on the grafted molecule (such a the molecule being an organosylane, or having a isocyanite group), or are confined to a single layer of molecule being attached to the surface of the cellulose to their design. The surface modification presented here offers the advantages of being both versatile in its graftable molecules, as SET-LRP is a versatile reaction and no significant adaptation to the grafting process was needed to adapt it from a monomer to another, and able to form a thicker layer around the cellulose fibres, as it is a polymerization reaction, and can therefore add more than a single monomer.

Some crucial work remains for the full development of this platform, with the testing of more SET-LRP-compatible monomer families to expand the pool of available monomers and a more in-depth study of the fibre-matrix compatibility, with both a wider range of matrixes (such as PLA of PP), and a thorough mechanical evaluation of the composites formed with various polymer-grafted cellulose fibres to rigorously demonstrate the high versatility of this platform as a mean to compatibilize cellulose fibres with a wide panel of polymer matrixes. This was left to future research.

## Author contributions

The manuscript was written through the contributions of all authors. All authors have approved the final version of the manuscript.

## Conflicts of interest

The authors declare that they have no conflicts of interest for the content of this article.

## Supplementary Material

RA-OLF-D6RA03700C-s001

## Data Availability

The data supporting this article have been included as part of the supplementary information (SI). Equation for the SAXS fitting, FTIR and NMR main peaks and interpretations. Supplementary information is available. See DOI: https://doi.org/10.1039/d6ra03700c.
