# Supplementary material for: SET-LRP and cellulose fiber: a versatile platform to tune the surface properties of cellulose
Source: RSC Adv. 2026 Jul 6;16(36):38165–73. doi: 10.1039/d6ra03700c (PMC13335733; doi:10.1039/d6ra03700c)
Supplement: Supplementary file 1 [file RA-016-D6RA03700C-s001.pdf]

Supplementary Information (SI) for RSC Advances.
This journal is © The Royal Society of Chemistry 2026

SET-LRP and Cellulose Fiber: a versatile platform to tune the surface properties of cellulose.

Enguerrand Barba[1], J. Benedikt Mietner[1], Dhanya Raveendrana[1], Benedikt Sochor[2,3], Sarathlal Koyiloth Vayalil[2,4], Stephan V. Roth[2,5], Julien R.G. Navarro[1]

Supplementary information:

1. SAXS Fitting: 2
ABS 3
CF-PBA@ABS 3
CF-PSA@ABS 4
CF-PDA@ABS 4
CF-PDEGEEA@ABS 5
CF-PSt@ABS 5
1. IR peak: 6
Cellulose[3–5]: 6
Modified Cellulose: 6
2. Reference 6

1. SAXS Fitting:

Fit was done using the extended Guinier-Porod model[1] with contributions for a gel[2]:

$$I(q) = F(q) + I_g e^{-\frac{(q-q_0)^2\theta^2}{2}} + \frac{I_l}{1+q^2\xi^2} + B$$

With F(q) being the extended Guinier-Porod model:

$$F(q) = \frac{I_0}{q^s} e^{-\frac{x^2R_g^2}{3-s}}$$

for $q < q_1$, otherwise:

$$F(q) = Dq^{-n}$$

With:

$$q_1 = \frac{1}{R_g}\left(\frac{(3-s)(n-s)}{2}\right)^{1/2}$$

$$D = I_0 e^{-\frac{q_1^2R_g^2}{3-s}} q_0^{(n-s)}$$

for continuity.

n is the Porod exponent, indicative of the particles' shapes

$\theta = \frac{1}{\sigma}$ with σ the variance of a Gaussian function, indicative of the size of aggregates

$\xi = \frac{1}{\gamma}$ with γ the scale factor of the Lorentz distribution, indicative of the correlation distance between adjacent fibers.

B is a flat background

After an initial estimation of the fitting values with a python program using the lmfit library, SASView was used for more accurate fitting, and to obtain the uncertainty on the fitted values. The background value of the composite sample was fixed to the value obtained for PVC alone. Finally, the individual contribution of the Guinier -Porod model, the Lorentzian and the Gaussian were plotted to estimate whether or not each part of the model had enough impact on the final curve to be considered relevant.

The following values were obtained from fitting:

ABS

Variable	Value	Error
I_0	4.57	9.99 e+07
R_g	416	1 e+08
s	1.36	4.3 e+6
n	3.7026	0.0009
I_g	0.084	0.035
x_0	0.125	0.009
σ	0.017	0.004
I_l	0.391	0.006
ξ	9.4	1.3
B	0	0.0689

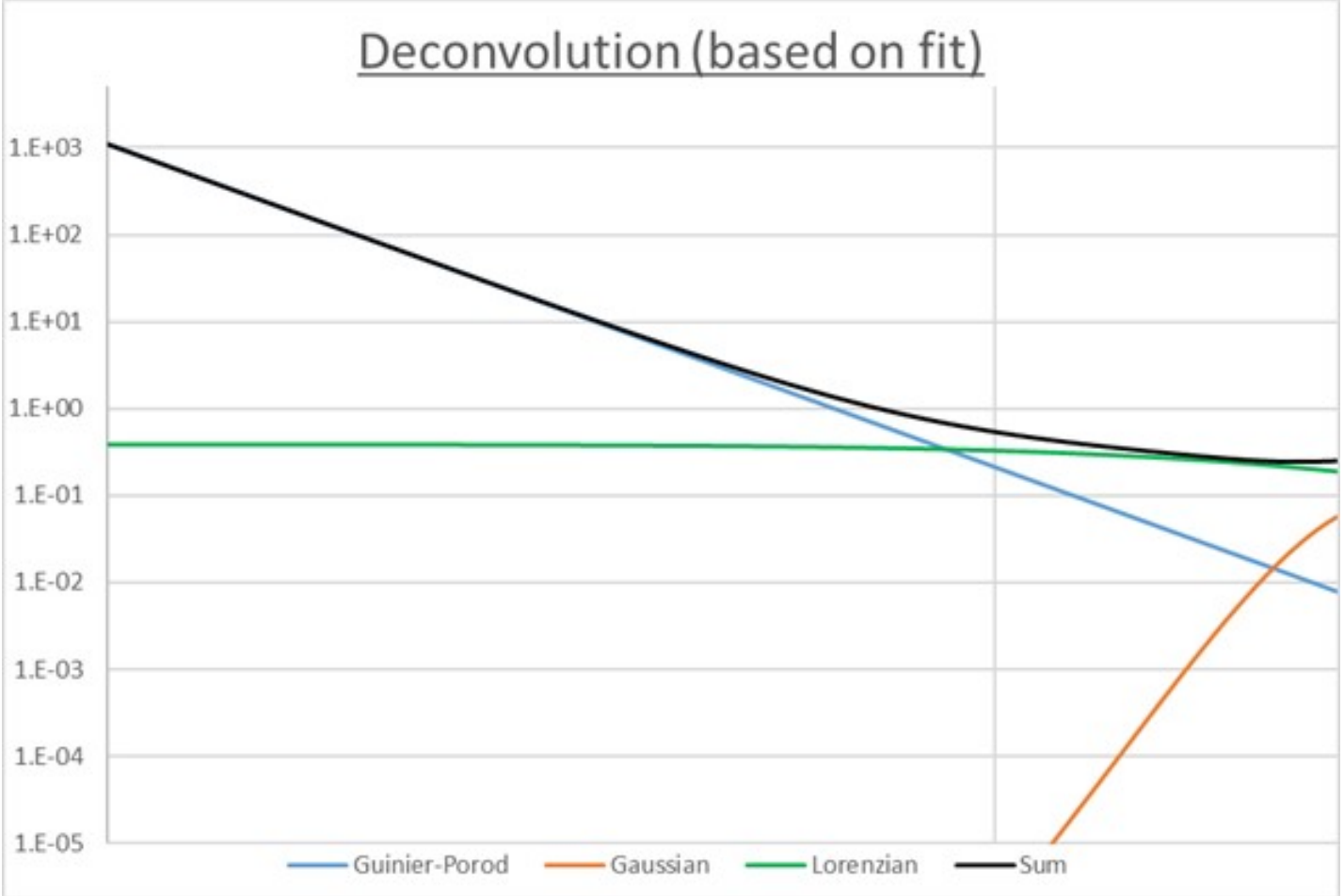

CF-PBA@ABS

Variable	Value	Error
I_0	175	$1\ 10^8$
R_g	320	$1\ 10^8$
s	0.37	$2.6\ 10^5$
n	3.61	0.003
I_g	0.66	1.4
x_0	0.286	0.22
σ	0.094	0.06
I_l	0.568	0.005
ξ	17.6	0.67
B	0	-

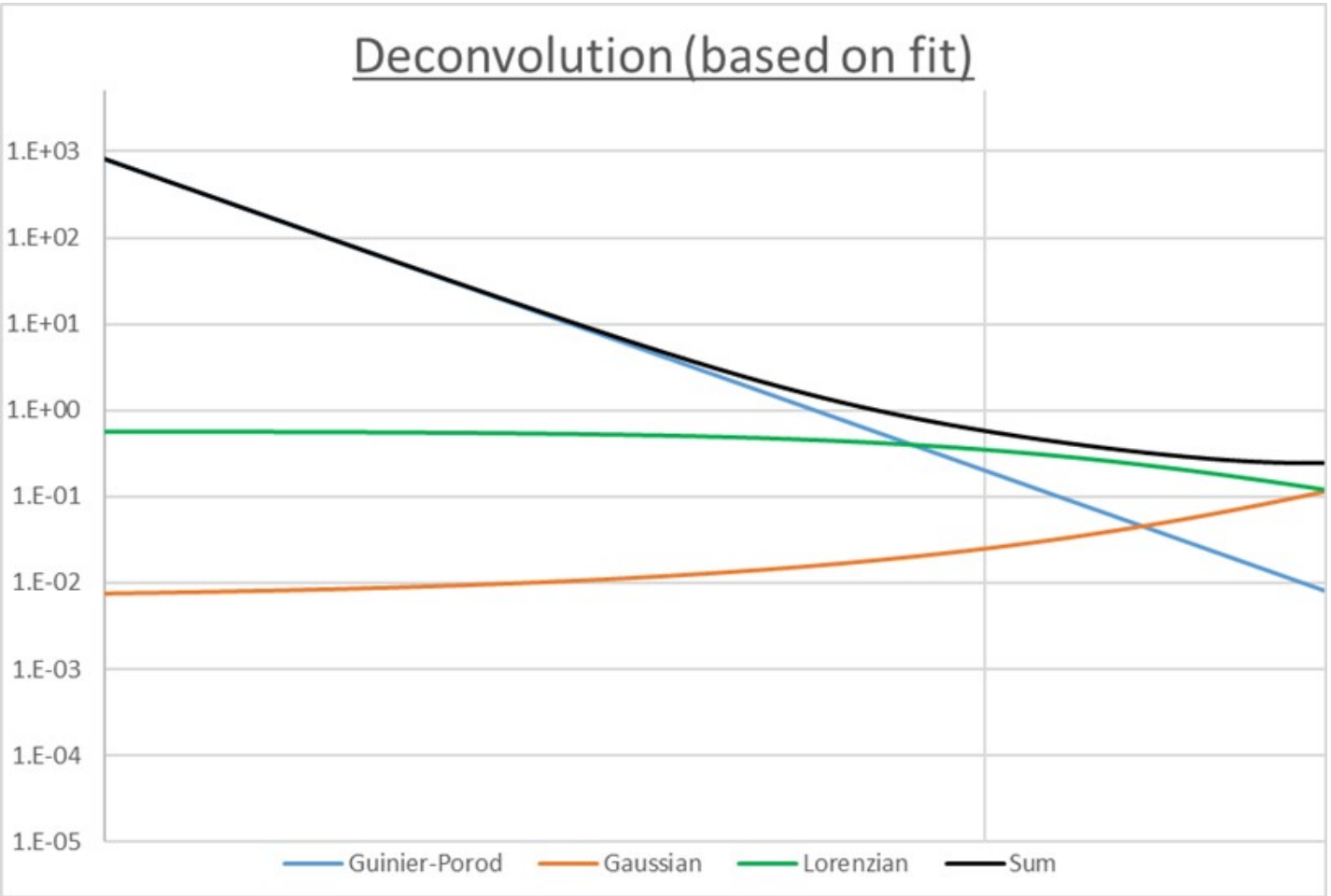
Deconvolution (based on fit)
1.E+03
1.E+02
1.E+01
1.E+00
1.E-01
1.E-02
1.E-03
1.E-04
1.E-05
Guinier-Porod
Gaussian
Lorenzian
Sum

CF-PSA@ABS

Variable	Value	Error
I_0	91	$1\ 10^8$
R_g	278	$1\ 10^8$
s	0.54	$3\ 10^5$
n	2.99	0.002
I_g	0.124	0.006
x_0	0.129	0.003
σ	0.0312	0.002
I_l	2.40	0.05
ξ	32.8	0.4
B	0	-

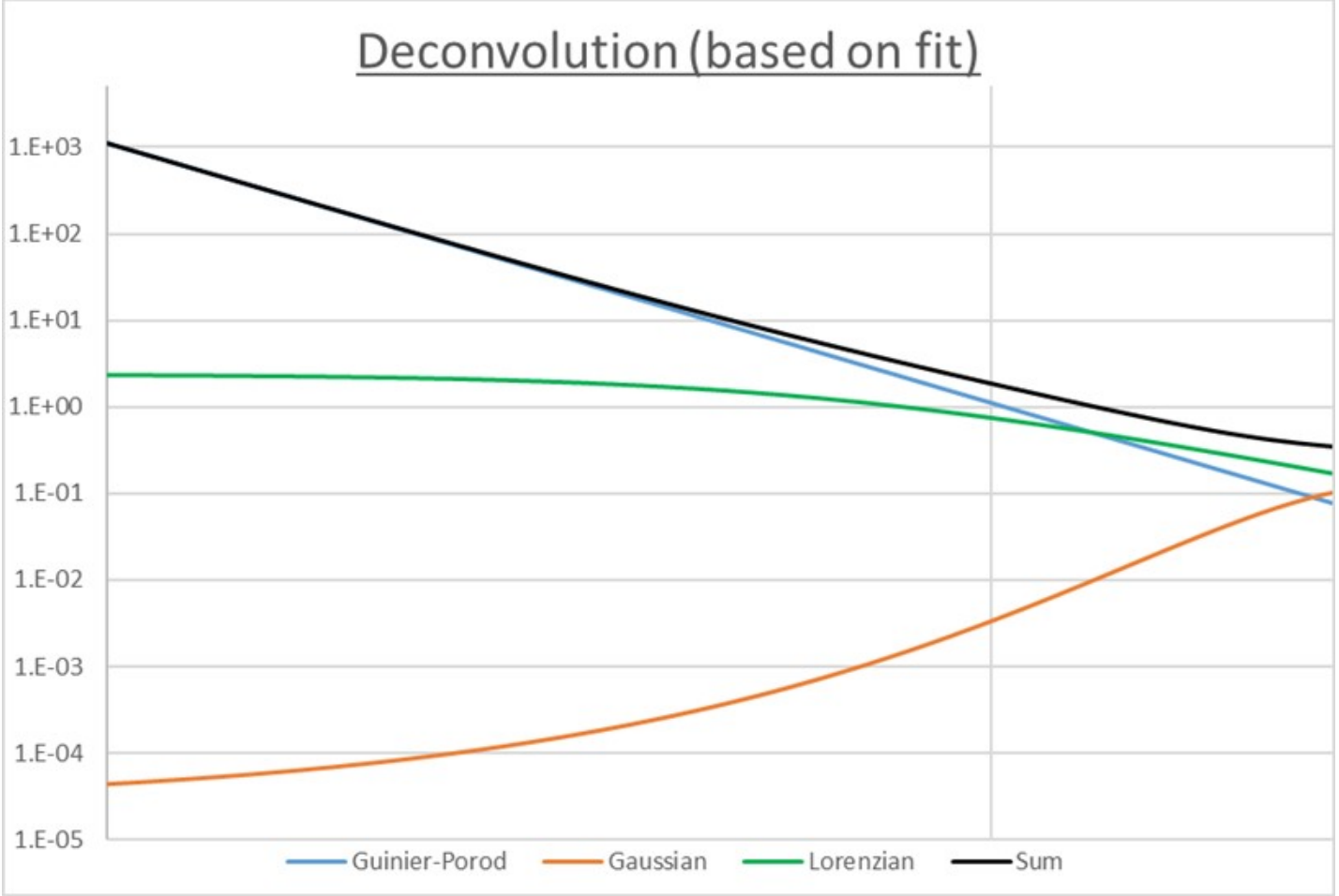

CF-PDA@ABS

Variable	Value	Error
I_0	496	$1\ 10^8$
R_g	383	$1\ 10^8$
s	0.39	$2\ 10^5$
n	3.23	0.008
I_g	0.182	0.001
x_0	0.1123	0.0007
σ	0.0392	0.0007
I_l	7.4	0.5
ξ	59	1.7
B	0	-

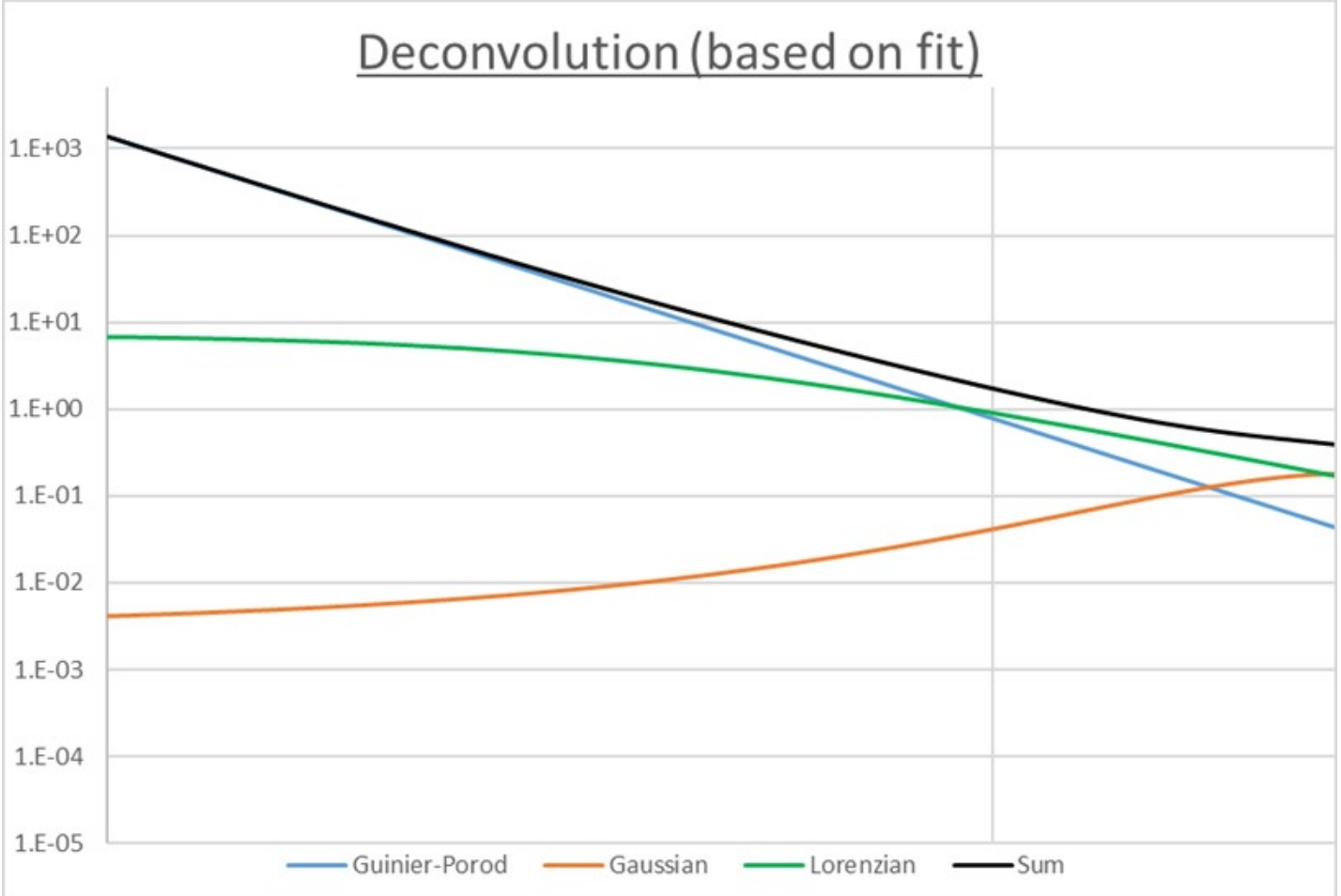

CF-PDEGEEA@ABS

Variable	Value	Error
I_0	37	$1\ 10^{8}$
R_g	209	$1\ 10^{8}$
s	0.68	$8\ 10^{5}$
n	3.780	0.009
I_g	0.28	0.68
x_0	0.178	0.015
σ	0.050	0.005
I_l	12.2	0.6
ξ	66.4	1.6
B	0	-

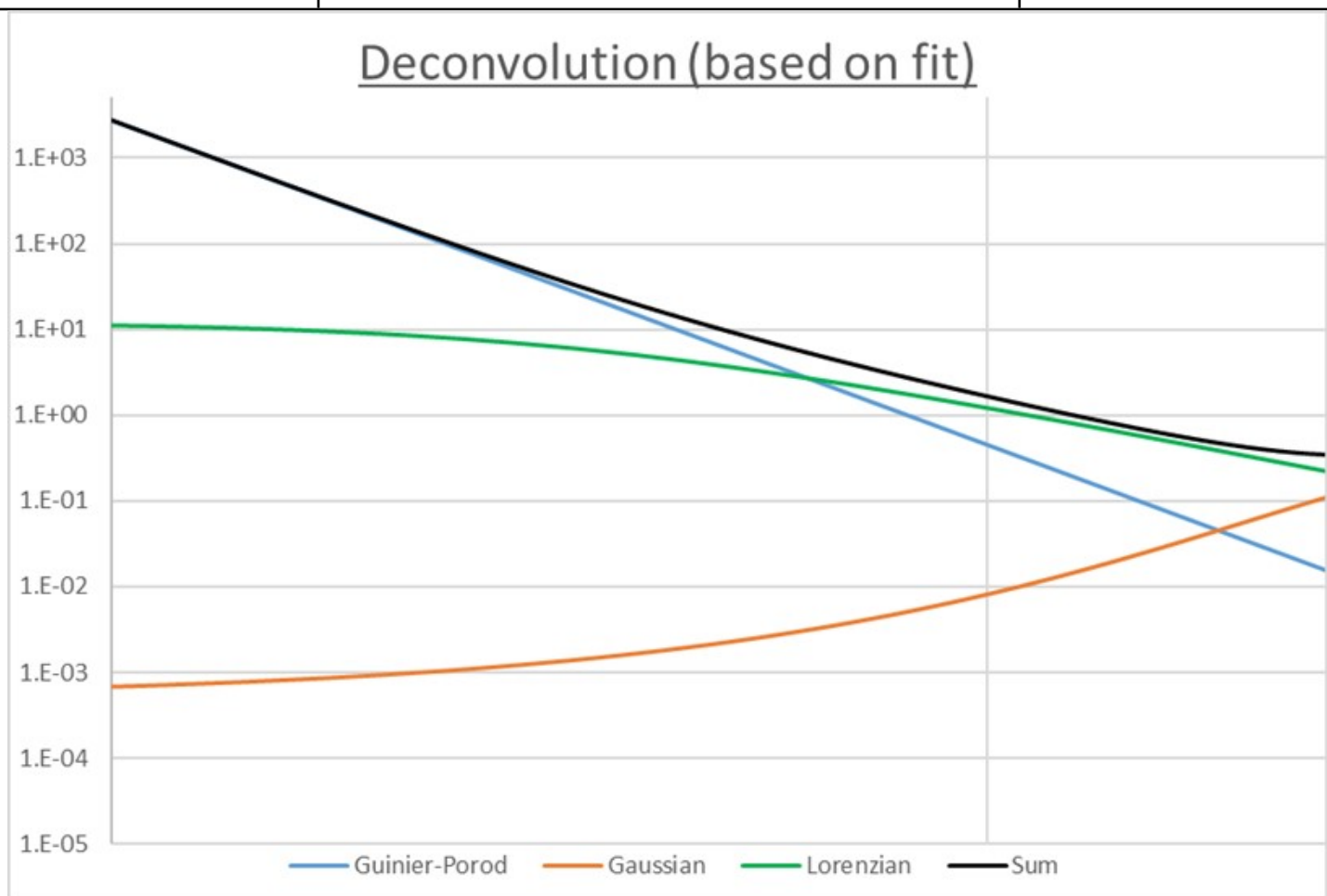

CF-PSt@ABS

Variable	Value	Error
I_0	6	4 107
R_g	162	9 107
s	0.88	1 106
n	3.395	0.004
I_g	0.21	0.09
x_0	0.15	0.01
σ	0.026	0.004
I_l	6.23	0.06
ξ	38.6	0.2
B	0	-

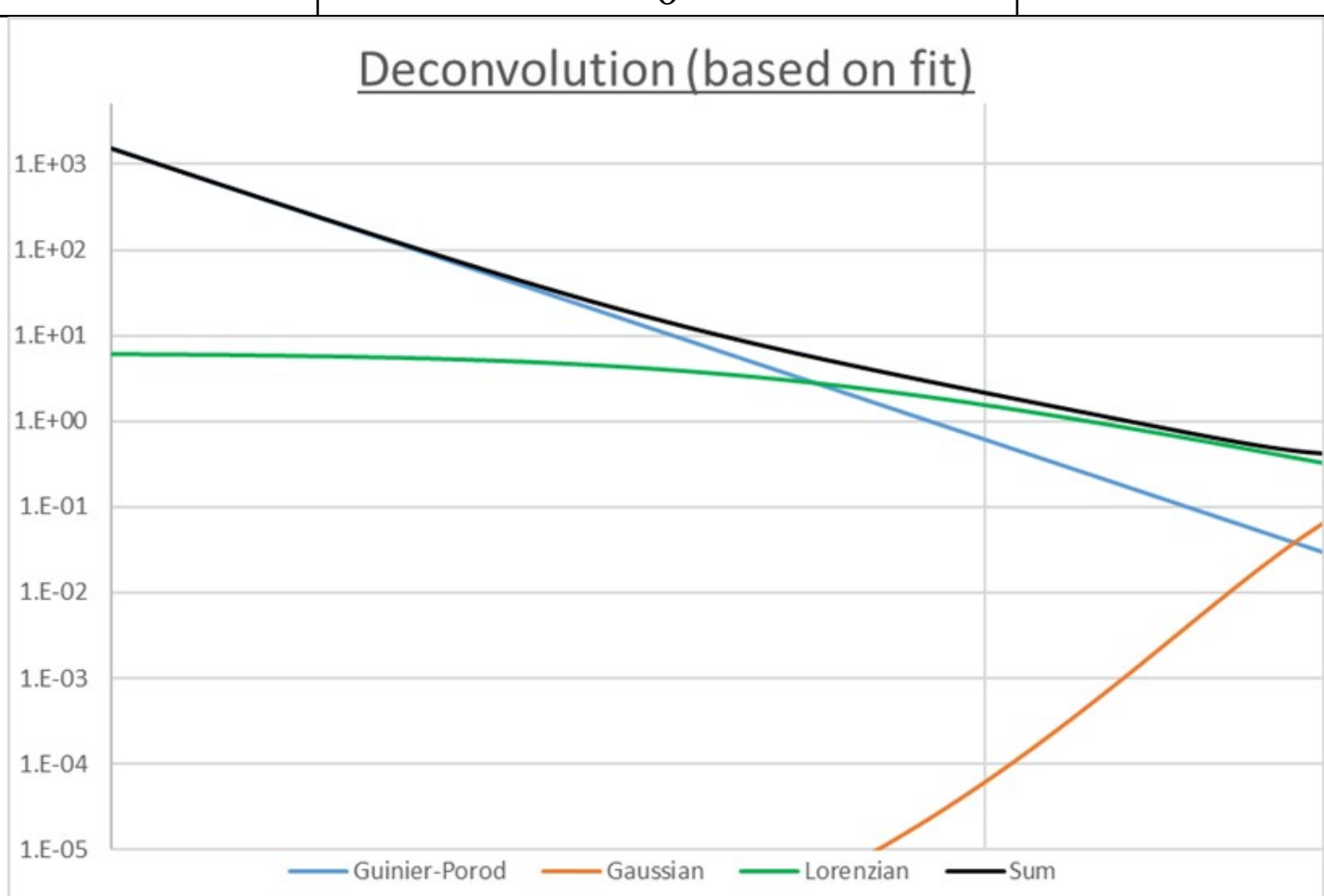

1. IR peak:

Cellulose[3–5]:

Wave number (cm^{-1})	Attribution
3340	O-H stretching
2870 - 2930	C-H stretching
1590 - 1650	Absorbed H_2O
(1160)	In-ring C-O-C stretching
(1030)	C-O stretching

Modified Cellulose:

Wave number (cm^{-1})	Attribution
(2950)	C-H stretching
(2930)	
(2850)	
(2830)	
(1770)	
(1722)	C=O stretching
698 (St)	Aromatic C-H out of plane bending
758 (St)	
1452 (St)	Aromatic C-C stretch
1493 (St)	
3021 (St)	Aromatic C-H stretch

2. Reference

1 B. Hammouda, A new Guinier–Porod model, *J Appl Crystallogr*, 2010, **43**, 716–719.

2 V. Guccini, S. Yu, M. Agthe, K. Gordeyeva, Y. Trushkina, A. Fall, C. Schütz and G. Salazar-Alvarez, Inducing nematic ordering of cellulose nanofibers using osmotic dehydration, *Nanoscale*, 2018, **10**, 23157–23163.

3 E. Singovszka, Characterization of Cellulosic Fibers by FTIR Spectroscopy for Their Further Implementation to Building Materials, *American Journal of Analytical Chemistry*, DOI:10.4236/AJAC.2018.96023.

4 C. Trilokesh and K. B. Uppuluri, Isolation and characterization of cellulose nanocrystals from jackfruit peel, *Sci Rep*, 2019, **9**, 16709.

5 O. S. Samuel, A. M. Adefusika, O. S. Samuel and A. M. Adefusika, in *Cellulose*, IntechOpen, 2019.
